# Recent Progress on Stability and Thermo-Physical Properties of Mono and Hybrid towards Green Nanofluids

**DOI:** 10.3390/mi12020176

**Published:** 2021-02-11

**Authors:** S.N.M. Zainon, W.H. Azmi

**Affiliations:** 1 Department of Mechanical Engineering, College of Engineering, Universiti Malaysia Pahang, Lebuhraya Tun Razak, Gambang, Kuantan 26300, Malaysia; sn.sharahzainon@gmail.com; 2 Centre for Research in Advanced Fluid and Processes, Lebuhraya Tun Razak, Gambang, Kuantan 26300, Malaysia

**Keywords:** nanofluids, green, stability, thermal conductivity, dynamic viscosity, thermo-physical properties

## Abstract

Many studies have shown the remarkable enhancement of thermo-physical properties with the addition of a small quantity of nanoparticles into conventional fluids. However, the long-term stability of the nanofluids, which plays a significant role in enhancing these properties, is hard to achieve, thus limiting the performance of the heat transfer fluids in practical applications. The present paper attempts to highlight various approaches used by researchers in improving and evaluating the stability of thermal fluids and thoroughly explores various factors that contribute to the enhancement of the thermo-physical properties of mono, hybrid, and green nanofluids. There are various methods to maintain the stability of nanofluids, but this paper particularly focuses on the sonication process, pH modification, and the use of surfactant. In addition, the common techniques to evaluate the stability of nanofluids are undertaken by using visual observation, TEM, FESEM, XRD, zeta potential analysis, and UV-Vis spectroscopy. Prior investigations revealed that the type of nanoparticle, particle volume concentration, size and shape of particles, temperature, and base fluids highly influence the thermo-physical properties of nanofluids. In conclusion, this paper summarized the findings and strategies to enhance the stability and factors affecting the thermal conductivity and dynamic viscosity of mono and hybrid of nanofluids towards green nanofluids.

## 1. Development of Nanofluids using Green Technology

The utilization of nanoparticles is expected to increase the efficiency of thermal systems in different industrial applications [1]. The efficiency of a thermal system can be improved by developing stable nanofluids with enhanced thermal conductivity. Increasing the efficiency of the thermal system will result in sustainable energy [2], a reduction in the size or cost of the thermal system [3], and reduction in harmful emissions [4]. Although nanofluids have been shown to be beneficial in various industrial applications, such as solar power systems [5,6], the threat to human safety and the environment posed by nanofluids have not yet been thoroughly investigated. Therefore, along with the development of nanofluids, interest in developing green nanofluids has also increased. The word “green” is often used to refer to any behavior-related approaches concerned with conserving the environment. According to Maksimović and Omanović-Mikličanin [7], green, environmental, or clean technology comprise methods and techniques that continuously evolve without risking the environment, conserving natural resources, and creating sustainable development methods. In fact, green nanofluids are categorized under green nanotechnology, which is one of the branches under green technology introduced by the government of Malaysia. According to Malaysia’s Ministry of Energy, Green Technology and Water (KeTTHA) [8], “green technology” can be defined as the development and application of products, equipment, and systems used to conserve the environment and resources, which minimize and reduce the negative impacts of human activities. Meanwhile, green nanotechnology represents an effort to utilize nature to eliminate or minimize the risk posed by the use of nanomaterials to the environment and humans and promote the replacement of existing products with more environmentally friendly nanoproducts [9].

The literature has demonstrated a modest contribution to the eco-friendly production of nanofluids. Recently, the suspension of nanoparticles synthesized from plants, fruits, and waste materials in the base fluid was proven to enhance the thermo-physical properties of nanofluids. Sadri et al. [10] developed an environmentally sustainable method that uses clove buds to treat covalently functionalized MWCNTs to study the heat transfer in the heat exchanger. The research reported a significant enhancement in the convective heat transfer with a negligible increase in friction factor. In a study conducted by Sadri et al. [10], the hydrothermal-assisted method was used to formulate the reduced graphene oxide nanofluids, and the results yielded a significant improvement in electrical and thermal conductivity. The rGO/water nanofluids also displayed almost unchanged viscosity and density with the increase in concentrations. The hydrothermal dehydration method is not only environmentally friendly, but it is also capable of producing nanoparticles in bulk and maintaining the purity of the products.

On the other hand, reducing agents and stabilizers are usually used to synthesize nanoparticles and improve the stability of the suspended nanoparticles by modifying the particle’s surface tension. However, materials such as sodium borohydride and cetyl trimethylammonium bromide (CTAB) intensify the toxicity of the environment, and this matter raises concern among researchers, especially in biomedical applications. Hence, many prior research considered bio-friendly reagents as an alternative to replace toxic reducing agents in the synthesis of nanoparticles, such as phytochemicals [11,12,13]. Phytochemicals are naturally extracted compounds found in plants that can be classified into several groups, such as natural gum, that act as a stabilizer. At the same time, isoflavones, organic acids, and catechins are also used as reducing agents [14].

Nune et al. [11] successfully synthesized gold nanoparticles sized in the range between 15 and 42 nm by mixing the Au ions with Darjeeling tea leaves. The stability of the suspension was further stabilized using Arabic gum. The suspension of nanoparticles was reported to be stable without agglomeration observed. Furthermore, Bahiraei et al. [15] investigated the performance and hydraulic characteristics of green silver nanofluids in a miniature heat exchanger. In the study, they biologically synthesized the silver nanoparticles using green tea leaf extract as a reducing agent. Sun et al. [16] also established a green preparation method to synthesize silver nanoparticles by mixing silver nitrate solution into green tea leaf extract. However, they reported the instability of silver nanofluids through zeta potential measurement at a high concentration of the tea extract. Stephen and Seethalakshmi [17] mixed silver nitrate solution with hesperidin that can be primarily obtained from citrus fruits, producing stable silver nanoparticles with a size range of between 20 and 40 nm.

Other than gold and silver nanoparticles, silica nanoparticles are also often used in the suspension of nanofluids due to their high specific surface area that allows more heat to be transferred. They can be greenly produced from the plant in bulk with low production cost. Recently, Ranjbarzadeh et al. [18] successfully produced silica nanoparticles sized less than 50 nm using the outer layer that coats rice, called rice bran. The rice bran underwent several processes including washing to remove the contamination on the rice bran before it was left to dry in the oven for 4 h at a temperature of 105 °C. Then, it was burnt in a furnace for 8 h at a temperature of 508 °C. The dispersion of the extracted silica with NaOH aqueous solution produces sodium silicate, and then neutralized by dilute sulfuric acid to precipitate the silica. After that, the solution was stirred for 24 h and left to age for 48 h to form a gel. Further processes such as filtering and washing by using water were done before freeze drying the gel. The suspension of silica nanoparticles in water displayed long-term stability for more than 6 months after preparation.

Apart from the nanoparticles themselves, conventional heat transfer fluids such as ethylene glycol (EG) and propylene glycol (PG) are often used as coolants, heat transfer agents, and anti-freeze in various industrial applications, such as automotives, electric power industries, and medicals [3]. EG is reported to be moderately toxic, with the oral lethal dose being low, LD_Lo_ = 786 mg/kg, for a human, and could cause harm upon ingestion. EG is colorless and odorless solution and has a sweet taste. Children or animals could mistakenly ingest the fluids due to its sweet taste. EG that enters the body is oxidized to oxalic acid, which is harmful and can affect the central nervous system. PG could replace EG, since it is safer to use and causes no harm to the body upon ingestion. However, both of these glycols are petroleum-derived products. Petroleum is a non-renewable energy source and is limited in supply, which is soon expected to diminish due to the high demand for energy. In addition, the extraction of petrochemicals from petroleum through burning pollutes the environment and causes global warming. Hence, it is vital to find a new source of glycol as an alternative to the EG or PG and other heat transfer fluids.

Recently, a new renewable bio-glycol (BG) produced by plants was used by Khdher et al. [19] as the base fluid in the formulation of Al_2_O_3_/BG nanofluids. BG is non-toxic to the environment and is domestically produced. In addition, BG is also a biodegradable glycol. The Al_2_O_3_/BG nanofluids were prepared using the two-step method without any addition of surfactant and showed long term stability. In their study, the thermal conductivity of BG-, EG-, and PG-based Al_2_O_3_ nanofluids was enhanced up to 17%, 9%, and 3.6%, respectively, at 30 °C for 1.0% volume concentration. In another study, Abdolbaqi et al. [20] successfully suspended Al_2_O_3_ nanoparticles in a mixture of water and BG at 60:40 and 40:60 ratios. Relative to the mixture of PG:water (W), the BG:water (W) mixture provided 7.5% enhancement in thermal conductivity at the same ratio. In addition, Abdolbaqi et al. [21] and Abdolbaqi et al. [22] also conducted a study on the thermal performance of colloidal suspensions of TiO_2_/BG:W and SiO_2_/BG:W nanofluids, respectively. In the study, the thermal conductivity was enhanced by up to 12.6% and 7.2% for the TiO_2_/BG:W and SiO_2_/BG:W nanofluids, respectively.

Currently, the ongoing research on green nanofluids is still in the early stages. The development of fully green nanofluids from non-toxic and renewable natural resources is hard to achieve since other factors such as compatibility, stability of the nanofluids, and availability of the resources need to be considered. A lack of knowledge and research on green nanofluids causes discrepancies in the literature. Most of the reviewed literature showed more interest in synthesizing nanoparticles using facile green techniques to replace the hazardous and expensive production methods. Meanwhile, a limited number of studies developed non-toxic stabilizers and reducing agents from natural resources such as plants and fruits. On the other hand, the alternative to replace EG and PG as the base fluid is still limited in the literature, and the introduction of green BG as the base fluid can be deemed as an opportunity to produce other types of green coolant. The implementation of green technology in the production of nanofluids promotes sustainability by using renewable natural resources, which will never deplete. However, the implementation is costly, and due to the limited study and lack of information [23], more obstacles are expected before green nanotechnology can be fully implemented. Hence, further investigation needs to be conducted in the future in order to fully adapt green technology in the production of nanofluids. Therefore, the present review is intended to highlight various approaches used by researchers in improving and evaluating the stability of thermal fluids, and thoroughly explores various factors that contribute to the enhancement of the thermo-physical properties of mono and hybrid nanofluids towards the development of green nanofluids.

## 2. Preparation of Nanofluids

**Figure 1:**
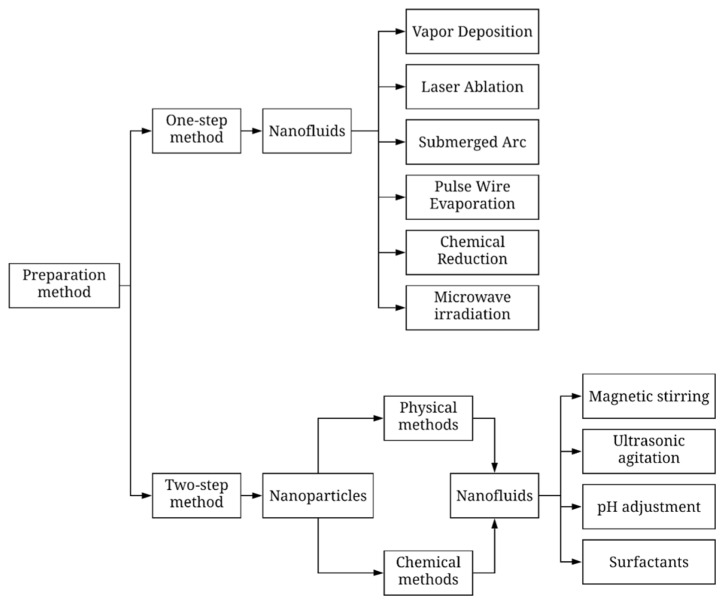
Nanofluids preparation methods.

The preparation of nanofluids is known to be an important stage that affects the stability and thermo-physical properties of the nanofluids. Hence, this section will discuss the two main methods that were commonly used by previous researchers in the preparation of nanofluids, namely the one-step method and two-step method, as shown in Table 1. The classification of nanofluid preparation methods is illustrated in Figure 1.

### 2.1. One-Step Method

The one-step method, also known as the single-step method, is typically used in small-scale productions. The one-step method is a process in which the synthesis of nanoparticles and dispersion of nanoparticles in the conventional base fluids are combined in a single step [37]. According to Ranga Babu et al. [38], highly stable and uniformly dispersed nanofluids can be obtained using this preparation method. There are various techniques to prepare nanofluids using the one-step method, including physical vapour deposition (PVD), the liquid chemical method, and vacuum evaporation onto a running oil substrate (VEROS) [4]. VEROS was first developed by Akoh et al. [39], where the nanoparticles are condensed from the vapour phase into a low pressure flowing fluids. Then, Eastman et al. [40] established another method that modified the VEROS technique. This new technique involves the metal vapour being condensed to nanoparticles and directly dispersed in the conventional base fluids.

In addition, the pulse wire evaporation (PWE) single-step method is also one of the most outstanding methods to prepare the nanofluids. Abdolbaqi et al. [22] stated that this technique required a high voltage pulse to be directed through a thin wire, then the wire will evaporate into plasma due to the non-equilibrium heating in a short period. This plasma is then condensed to nano-sized powder upon interaction with inert gas, such as Ar or N_2_, and combined with the nanofluids that are poured into an exploding bottle installed in the PWE device to form hybrid nanofluids. Munkhbayar et al. [41] also used this method in their research to prepare Ag-MWCNT/water hybrid nanofluids. Purified MWCNT nanoparticles that had previously undergone chemical treatments were transferred to the exploding bottle installed in the PWE setup. The Ag nanoparticles were then mixed with the base fluids and MWCNT/water nanofluids inside the PWE instrument. In addition, the one-step method eliminates multiple steps that are usually used with the two-step method, such as storing, drying, dispersing, stirring, and sonication, which ultimately help to minimize the agglomeration of the nanoparticles [37].

### 2.2. Two-Step Method

The two-step method separates the production of nanoparticles from the nanofluids’ preparation. In this method, the nanoparticles are first synthesized using chemicals or physical methods and then dispersed into the base fluids. Nowadays, nanoparticles are commercially available on the market and can be purchased in powder or liquid form, hence increasing the employment of the two-step method for nanofluids preparation. This is the most economical method to prepare nanofluids on a large scale. However, the challenge of using this preparation method is to obtain a stable suspension of nanofluids. Due to their high surface area and surface activity, the nanoparticles tend to agglomerate before they are entirely dispersed in the base fluids. The particle agglomeration will finally cause the separation between nanoparticles and base fluids, forming sedimentation [42,43]. The sedimentation in nanofluids causes clogging of the micro channel and a decrease in the thermal conductivity [44].

Various techniques were employed to reduce the agglomeration of particles and increase the stability of the nanofluids by using physical and chemical treatment methods to modify the surface properties [45]. Physical treatment methods are used, such as magnetic stirring, ultrasonic agitation, homogenization, and ball milling [46]. Both magnetic stirrer and sonication devices are commonly used by researchers, such as Nabil et al. [47], Zhao et al. [48], and Hamid et al. [49]. For chemical treatment methods, pH adjustments and the addition of the surfactants were employed to enhance the stability of the nanofluids [46]. According to Manna [45], the two-step method is suitable for the preparation of oxide nanofluids, but is less suitable to prepare metallic nanofluids. There is a probability that the nanoparticles would oxidize, thus using the two-step method for metallic nanoparticles is not preferable [43]. However, Wang and Mujumdar [50] stated that the two-step method could be used to prepare almost all types of nanofluid. Akilu et al. [26], Ahmed et al. [32], Moldoveanu et al. [29], Graves et al. [35], and Asadi et al. [36] successfully prepared nanofluids using TiO_2_-CuO/C, TiO_2_, Al_2_O_3_-SiO_2_, Cu, and CuO-TiO_2_ nanoparticles in various types of base fluid and maintained good dispersion stability.

## 3. Stability of Nanofluids

The superiority of nanofluids as heat transfer fluids has already been established by various researchers [51,52,53,54,55]. However, to be defined as an outstanding heat transfer fluid, nanofluids must have good stability. Stability is a critical component to improve the heat transfer capability of the nanofluids [56]. Nanofluids are said to be stable when they do not agglomerate and show slow particle settling. According to Che Sidik et al. [57], nanofluids tend to agglomerate and may lose their ability to transfer heat efficiently. Ghadimi et al. [58] said that the particle clustering due to the strong van der Waals force between nanoparticles is one of the main challenges in the formulation of homogenous suspensions. It is therefore vital to study the dispersion stability of nanofluids intensively. This section will discuss several methods used in the literature to enhance and evaluate the stability of nanofluids.

### 3.1. Stability Improvement Methods

Past studies have used many approaches to enhance the stability of nanofluids, such as ultrasonic agitation and surfactant addition [59]. Sonication has been used in many industrial applications, such as food and beverage technology, mineral processing, medical scanning, ultrasonic therapy, environmental decontamination, and also commonly in nanotechnology applications to enhance the stability of nanofluids [60]. The sonication process is a process of applying sound energy to agitate particles in the sample [61]. Ultrasonication is achieved by applying frequencies that are more than 20 kHz [61]. This process can be carried out by using a sonication bath or probe. The sonication bath transfers ultrasonic waves through the water to the sample, whereas the probe is placed directly into the sample. The sonication process using a probe is considered to work better than a sonication bath due to its high localized intensity. However, due to the potential contamination through the tip of the probe, erosion of the titanium probe tip after continuous usage, and the difference in the immersion of the probe, the sonication bath is favored over probe-type sonication [62]. In addition, the simultaneous sonication process can take place at the same time in the sonication bath, thus reducing the preparation time. The samples are obtained under similar conditions and behave comparably to nanoparticles’ dispersion behavior. During the preparation, the nanofluids are subjected to the sonication process to alter the morphological traits of nano-sized particles and to break up the agglomeration of colloidal suspension. The agglomeration of particles not only decreases the overall effective thermal conductivity, but also may result in the clogging of the system [63].

While it is proven that ultrasonication can improve the stability of nanofluids, there is no standard sonication time available in the literature. The sonication time may have a different effect on different types of nanofluids. According to Afzal et al. [61], the sonication time is different for each nanofluid. In order to determine the optimum sonication time, several factors need to be considered, including the type of sonication device, power, frequency, concentration of nanofluids, and base fluids. Recently, Nabil et al. [64] studied the effects of different sonication times on the stability of TiO_2_-SiO_2_/water:EG hybrid nanofluids by observing their absorbance ratio using ultraviolet-visible spectroscopy. They found that the nanofluids with longer sonication time maintained a high absorbance ratio after some time. However, the absorbance ratio for nanofluids with 2.0 h sonication time was observed to be lower than that of nanofluids with a sonication time of 1.5 h. Similarly, Chen et al. [65] investigated the effects of different sonication times on the thermal conductivity of Al_2_O_3_/liquid paraffin nanofluids at various concentrations and temperatures. They observed an increase in relative thermal conductivity with up to 3.0 h of sonication time, and then reduced at 4.0 h of sonication time. The study reported that the decrease in the relative thermal conductivity for nanofluids with 4.0 h of sonication was probably due to the bonding separation between the nano additives and surfactant. Then, they narrowed down the sonication time to 2 h 45 min, 3 h 15 min, and 3 h 45 min. Based on the observation, they found that the sample preparation with up to 3 h 15 min sonication time performed with the highest thermal conductivity for the Al_2_O_3_/liquid paraffin nanofluids.

Kole and Dey [66] performed an investigation on the cluster size of ZnO/EG nanofluids at different sonication times ranging between 4 and 100 h. The study observed a decrease in the size of the particle cluster as the sonication time increases to 60 h, and after that, the cluster size increased. ZnO/EG nanofluids with 60 h sonication times are reported to be stable without any visible sedimentation for up to 30 days. Mahbubul et al. [67] studied the stability of Al_2_O_3_/water nanofluids at different sonication times and amplitudes by measuring the zeta potential. The maximum zeta potential up to 58.4 mV was achieved by subjecting the Al_2_O_3_/water nanofluids to 3 h sonication at 50% amplitude, and further sonication of the sample decreased the value of the zeta potential. However, at a 25% amplitude, a longer sonication time is needed for the Al_2_O_3_/water nanofluids to achieve maximum zeta potential of 57.5 mV, which is 5 h. They also reported a reduction in particle agglomeration with the increase in sonication time and amplitude. The paper suggested that a lower amplitude requires a longer time to de-agglomerate the nanoparticles. In another study, Mahbubul et al. [68] explored the effects of sonication time on TiO_2_/water nanofluids. The samples were subjected to the ultrasonication process for 0, 30, 60, 90, 120, 150, and 180 min. The average cluster size was observed to decrease when they increased the ultrasonication time. However, the average particle shows insignificant difference in size for ultrasonication time between 150 and 180 min. Prior investigations indicated that the optimum ultrasonication time to produce a good dispersion and stability is unique for each nanofluid.

In addition, the stability of nanofluids can also be enhanced by adjusting the pH. Ghadimi et al. [58] stated that the stability of nanofluids could be associated with the electro-kinetic properties. The stability of the nanosuspension can be improved when there is a strong repulsive force between the particles, and this can be achieved by modifying the pH [69]. Kamalgharibi et al. [70] examined nanoparticles with good dispersion in the base fluids and that possessed high surface charge densities. The nanoparticles are capable of creating strong repulsive forces to stabilize the nanofluids. Witharana et al. [71] stated that the optimum pH should be higher or smaller than the isoelectric point (IEP), which can be observed when zeta potential is around zero. In their study, the TiO_2_/W:EG and TiO_2_/W:PG nanofluids were prepared at different pH values. Then, the stability of the nanofluids was evaluated by measuring their zeta potential value. The IEP for TiO_2_/W:EG nanofluid and TiO_2_/W:PG nanofluid was obtained at a pH of 4.7 and 6.8, respectively. TiO_2_/W:EG nanofluid demonstrated a maximum zeta potential value at a pH ranging from 6.2 to 7.8, which is far from the IEP. Both samples were kept under observation for two months and were found to be stable with minimal sedimentation.

Similarly, Choudhary et al. [72] prepared Al_2_O_3_/water nanofluid at different concentrations with adjusted pH values from 2 to 11. They reported that the IEP for all concentrations of Al_2_O_3_/water nanofluids is found at 8.6. Subsequently, the maximum absolute zeta potential was observed in the acidic region (pH = 3). They also explained that when a pH value is bigger than the IEP, the ionic strength of the solution will increase, while the zeta potential value decreases in the negative direction. Likewise, when pH values are smaller than the IEP, the zeta potential will show increments in the positive direction caused by the reduction in ionic strength in the sample. Kazemi et al. [73] prepared the SiO_2_/water and G/water nanofluids at different pH values of 3, 6, 9, and 12 and evaluated the stability of the samples using visual observation. They reported that the SiO_2_/water nanofluids were stable at all pH values, especially at pH higher than 3, while G/water nanofluids demonstrated better stability at higher pH values. Leong et al. [74] found in their study that there is an increment in the thermal conductivity of Cu-TiO_2_/EG:W hybrid nanofluids as the pH value increases. The hybrid nanofluids achieved a maximum increase in thermal conductivity at pH = 7; however, a further increase in pH reduces the thermal conductivity of the hybrid nanofluids. Akilu et al. [26] found that the IEP for TiO_2_-CuO/C:EG hybrid nanofluids is in the acidic region; hence the nanofluid samples were adjusted to be more basic (pH = 10) by adding NaOH solution. A high zeta potential value was observed for all the samples, which indicates that the colloidal suspension has good stability. In many previous investigations, both of these techniques were shown to reduce the agglomeration of particles and further enhance the stability of nanofluids.

### 3.2. Stability Evaluation Methods

The development of technology has allowed researchers to evaluate the stability of nanofluids in various ways. Since stability can affect thermal performance, stability needs to be adequately evaluated using different methods. The common techniques used by researchers in the literature include visual observation, particle characterization using transmission electron microscopy (TEM), scanning electron microscopy (SEM), and field-emission scanning electron microscopy (FESEM), zeta potential analysis, and ultraviolet-visible spectroscopy. These methods will be discussed in detail in the next section.

#### 3.2.1. Visual Observation

The visual observation method or sedimentation method has been widely used in this field of research to observe sedimentation in a sample nanofluid for a specified period. The solid particles dispersed in the base fluid do not dissolve but remain suspended in the fluid, floating randomly. However, the particles suspended in the base fluid tend to settle out of the fluid due to several factors, such as gravity, centrifugal acceleration, and electromagnetism. Dispersion stability is essential to prolong the shelf life of the nanofluid sample under certain conditions and maintain its properties and quality over time. The shelf life of the sample is measurable and can be seen by the naked eye through prolonged observation. The sample is said to be stable for a certain period based on its sedimentation level or height, which is usually measured in millimetres (mm).

Hamid et al. [75] evaluated the stability of TiO_2_ water/ethylene glycol-based nanofluids by measuring the thickness layer of nanofluid. After two weeks, the separation thickness layer of 5 and 3 mm appeared for the sample at volume concentrations of 0.5% and 0.7%, respectively. Then the sample was left to rest and observed again after seven months of preparation. All samples from 0.5% to 1.5% volume concentrations showed a very thin supernatant, especially at high volume concentrations of 1.5%, which is 3 mm. Similarly, Khdher et al. [19] dispersed Al_2_O_3_ nanoparticles into water and ethylene glycol mixture and quantitatively expressed the stability of the sample through the sedimentation ratio, which can be calculated with Equation (1). They reported that the samples were stable for 30 days, with a slow sedimentation rate in the first ten days.

(1) $${\mathrm{Ratio}}_{\mathrm{sedimentation}}=100\%-\left(\frac{h_{o}-h_{i}}{h_{o}}\right)\times 100\%,$$

where $h_{o}$ and $h_{i}$ represent the original height of homogenous nanofluids and height of sedimentation in time, respectively.

The most straightforward technique to observe the change in a sample of nanofluids was undertaken by photographing the sample and was practised by many investigators, such as Azmi et al. [76], Islam et al. [77], Hamid et al. [78] and Ranjbarzadeh et al. [18]. One can photograph the sample as often as possible until the separation layer appears in the fluids. The time for the sedimentation to appear is a measure of the stability condition and should be recorded. There is no standard time for the sample to show the separation layer, since it can be influenced by many factors, such as size and shape of particles, preparation methods, and concentration of the sample.

#### 3.2.2. Micrograph and Imaging Observation

The interest in studying the dispersion of nanomaterials in conventional thermal fluids has increased over the years due to its great potential in enhancing the thermo-physical properties of the fluids. However, often the characterization of nanoparticles dispersed in base fluids is inadequately reported by researchers. This may lead to limited or inaccurate analysis with conclusions concerning particle properties and behaviors [79]. The importance of the characterization of particles in determining good dispersion stability is to establish a standard dispersion method and its effects on thermo-physical properties. Due to the rapid development of technology, various methods have been developed to study the physical and chemical characterization of nanoparticles and dispersion behavior evaluation. The techniques mentioned herein are transmission electron microscopy (TEM), scanning electron microscopy (SEM), and field-emission scanning electron microscopy (FESEM). TEM, SEM, and FESEM are straightforward methods. These methods are frequently used by the investigators in observing the state of dispersion, particle size, and shape within nanofluids. Most researchers analyzed the image in acquiring particle size distribution to relate with the stability of the nanofluids.

Both TEM and SEM use electron beams to study the characterization of the sample. However, as the name implies, TEM operates by transmitting the electron beam through the ultra-thin samples and generate 2D images. On the other hand, SEM scans the surface of the sample using scattered electrons and is able to produce 3D images of the sample through secondary electrons emitted from the surface caused by primary electron beam excitation. Yu et al. [80] stated that the morphological structure of nanofluids, changes in shape and structure of particles could be analyzed using SEM, but compared to TEM, SEM has much lower resolution. TEM provides a high-resolution image of nanoscale particles with 5,000,000× magnifying power [81]. The penetration of the electron beam through the sample has allowed TEM to be used in measuring the nanoparticle size, grain, and crystallite size in the nanocomposite. Meanwhile, SEM provides limited functions that only show the morphological surface of the particles and are not capable of measuring the nano-sized particles. These reasons have made TEM the preferred choice by researchers for particle characterization over SEM. FESEM possesses the same features and functions of SEM but is applicable at a high resolution. In addition, another obvious difference between SEM and FESEM can be seen in the type of emitter. The emitters in TEM and SEM are a thermionic emitter and field emitter, respectively. In comparison, the source of the electron in FESEM is much brighter, and its beam size is much smaller, which leads to higher magnification power. Dhinesh Kumar and Valan Arasu [82] mentioned that the image provided by FESEM is clearer and less electrostatically distorted, with its spatial resolution reduced to 1.5 nm, which is three to six times better compared to conventional SEM.

The application of these methods in studying the stability of nanofluids has provided a useful insight on the size, shape, and orientation of particles, and morphology of stabilized nanofluids after preparation. Chakraborty et al. [83] studied the surface morphology of raw TiO_2_/water and TiO_2_/water nanofluids using SEM. The authors used a small amount from the sample and deposited a thin layer of powdered nanoparticles on a carbon grid and dried it by placing the deposited sample under a mercury lamp. Since the sample needs to have a conductive surface, a conductive layer of gold metal was used to coat the sample before starting the characterization process using SEM. Besides ensuring the sample surface is electrically conductive, it is also crucial to keep the sample dry to obtain functional image analysis. The drying process of the sample should be handled carefully to avoid aggregation that can result from this process. Yu et al. [80] mentioned that during the drying process for oil-based nanofluids, it is very challenging to acquire a high microscopic sample due to the adsorption of oil onto the nanoparticle surfaces and causing strong discharging effects during characterization using SEM. For TEM, the image produced could get blurry.

Chakraborty et al. [83] also conducted the characterization using TEM to study the particle size distribution of raw and treated TiO_2_ nanoparticles. From the observation of SEM image analysis, the raw TiO_2_ nanoparticles appear agglomerated, while synthesized TiO_2_ nanoparticles appear smaller in size after being subjected to a long sonication process. Meanwhile, TEM images were also analyzed by Chakraborty et al. [83] and they found that both raw and treated nanoparticles mostly exist in spherical forms, and others vary from hexagonal to square and rectangular shapes. The nanoparticles before treatment are above 100 nm in size, but after treatment, this becomes less than 100 nm, with 95 nm being the average size. Khdher et al. [19] had used FESEM at 3,000,000× magnification power to evaluate the characterization of Al_2_O_3_/W:BG nanofluids at different base fluid mixture ratios of water and biogylcol (W:BG). The median particle size observed from the FESEM analysis is 13 nm, and the nanoparticles are mostly spherical. Similarly, Chiam et al. [84] also studied the characterization of Al_2_O_3_/W:EG nanofluids, but dispersed in different mixture ratios of water and ethylene glycol (W:EG). They obtained the same average size of 13 nm for Al_2_O_3_ nanoparticles, and the nanoparticles appeared to be spherical.

#### 3.2.3. Zeta Potential Analysis

Zeta potential (*ξ*-potential) is a crucial method that acts as a quantitative indicator for the dispersion stability of nanofluids. It is measured in millivolts (mV) and can be defined as the potential difference between the surface of nanoparticles and the stagnant layer of fluids attached to the particles. In other words, it is a magnitude that represents the degree of electrostatic charge between nanoparticles suspended in a liquid. Zeta potential evaluation is performed to enhance dispersion, suspension, and emulsion formulation and, at the same time, allow researchers to explore the source of dispersion and aggregation. Zeta potential reflects the degree of stability of the nanofluids, thereby playing a significant role in the nanocomposite research topic.

When particles are dispersed in fluids, there will be attraction and repulsion between particles that are influenced by particle distance and total interface energy. This total interface energy is the summation of van der Waals interaction and electrostatic repulsion, which is related to the Derjaguin, Landau, Verwey, and Overbeek (DLVO) theory. The theory quantitatively described the clustering of the aqueous dispersion and the force of interaction between charged surfaces through a liquid medium. This theory uses zeta potential to explain the development of repulsive force when the ionic atmosphere of two particles overlap as they approach each other. Zeta potential is crucial in determining the isoelectric point (IEP) and finding the optimum pH value for nanofluids. Krishnakumar et al. [85], in their paper, defined the isoelectric point as the value of pH at which certain particles or surfaces carry no net electrical charge.

Mukherjee and Paria [86] stated that high absolute zeta potential indicates high stability of the colloidal suspension, whereas, with a lower zeta potential, the probability of the colloids to coagulate is increased. This happens because the force of attraction between particles exceeds the repulsion force. As reported by Lu and Gao [87], colloids with zeta potential value between −11 and −20 mV approached agglomeration, while those with zeta potential values between −41 and −50 mV showed excellent stability. Similarly, Mukherjee and Paria [86] also mentioned in their review that stabilized nanofluids have zeta potential values between 40 and 60 mV. The colloid stability behavior of particles based on zeta potential value is summarized in Table 2.

It is known that one of the factors affecting zeta potential value is the pH value of nanofluids. A positive or negative zeta potential value can be changed by controlling the pH values. The addition of alkaline or acid solution into colloids alters the surface charge of nanoparticles caused by the adsorption of H^+^ ions or OH^−^ on the surface of nanoparticles. In the acidic region, a positive value of zeta potential was obtained since more H^+^ ions were adsorbed. Likewise, in the basic region, more OH^−^ ions were adsorbed, causing the zeta potential to decrease to a negative value. However, at a certain pH, the molecule or particle may carry no electrical charge, where the zeta potential value obtained is zero. The stability of nanofluids is always linked with pH and potential value because if the pH value moves away from the IEP, then the nanofluid is considered stable. This is relevant to the stability behavior portrayed in Table 2, where at zeta potential = 0, the colloids will coagulate and have the least stability.

#### 3.2.4. Ultraviolet-Visible Spectroscopy

Ultraviolet-visible spectroscopy, or its short name, UV-Vis spectroscopy, is a method used to evaluate the stability of nanofluids by measuring the absorbance of fluid at one time or over time. This method has been used as a modern approach to qualitatively determine the dispersion stability of colloids [89]. The basic concept of this method can be understood by using the Beer–Lambert law, which states that absorbance is directly proportional to the concentration of the solution or fluid tested. The UV-Vis measurement device consists of a light source that passes through a monochromator that alters the wavelength according to the input and passes the light beam through the pre-aligned sample cell. Then, the detector will detect the beam and convert it into an electrical signal in photocells and transfer it to the amplifier.

This method was employed by many researchers to evaluate the stability of nanofluids, usually by observing the absorbance of nanofluids at a certain period. The nanofluids are said to be stable when the particles in the dispersed fluids remain floating and not precipitated at the bottom, so more light is absorbed by the particles, producing a high absorbance rate. Sadeghi et al. [90] studied the stability of alumina nanofluids at different sonication times of the UV-Vis spectrum. They found the maximum peak of absorbance was at *λ* = 190 nm. The authors then investigated the absorbance of the nanofluids over 30 days at *λ*_max_. They observed that the absorbance gradually decreased over time. Alumina nanofluids at 3% volume concentration showed the highest absorbance and maintained its stability for up to 30 days. This method was reported as a reliable method. The data agreed well with the data obtained from the dynamic light scattering (DLS) method. UV-Vis absorption spectra for SWCNT nanofluids were investigated by Yu et al. [91] in the wavelength between 320 and 1350 nm and two maximum peaks at the wavelengths of 976 and 551 nm were found. A summary of the available studies on stability improvement and evaluation techniques is tabulated in Table 3.

## 4. Thermo-Physical Properties of Nanofluids

The thermo-physical properties of nanofluids, namely thermal conductivity, dynamic viscosity, density, and specific heat, change with temperature and volume concentrations. These properties have been studied by various researchers in different applications to evaluate the characteristics of heat transfer fluids and determine the optimum conditions for heat transfer fluids to work effectively in the system. Nanoparticle dispersion behavior and heat transfer performance at different operating conditions can only be evaluated after establishing the thermo-physical properties of the nanofluids. In the previous investigations by various investigators, several factors that affect the thermo-physical properties of nanofluids, such as base fluids, types of nanoparticles, temperature, particle loading, and hybrid composition ratio, were identified [29,54,98]. However, the degree of contribution to which these factors influence the thermo-physical properties of nanofluids remains unclear, thus leading to numerous investigations utilizing various types of nanoparticles.

Conventional heat transfer fluids such as water, ethylene glycol (EG), propylene glycol (PG), and engine oils are commonly used in cooling systems. The function of these fluids is to transfer the heat from the device in the system. This can prevent overheating to avoid underperformance and damage to the system. These fluids were used for a long time. Nowadays, the advances in technology and compactness in the system require new fluids with better performance to overcome the limitations of conventional fluids in thermal properties. In a recent study by Yasinskiy et al. [99], they reported that suspended nanoparticles in a host fluid, namely nanofluids, showed positive enhancement in thermal conductivity, and subsequently better heat transfer performance than conventional fluids. However, mono nanofluids could not provide all the positive characteristics which are mandatory for a specific purpose, and lacked some of the rheological and thermal properties [4]. Hence, the most recent development in nanofluids research led to the invention of new nanofluids with two or more types of nanoparticles dispersed in a base fluid. These kinds of nanofluids have better characteristic features to overcome the limitation of mono nanofluids due to the combination and exchange of different constituent materials [100,101].

The disadvantages of nanofluids’ rheological properties are due to the greater increment in viscosity, which is considered one of the drawbacks in heat transfer applications. Furthermore, the addition of nanoparticles into base fluids will also alter the density and specific heat of nanofluids. The density and specific heat of nanofluids were estimated using existing mixture ratios from the literature. The density of nanofluids is higher compared to conventional fluids [102], thus the molecules will be closely packed. Hence the heat transfer due to conduction will be improved due to rapid intermolecular vibration between nanoparticles. However, the specific heat of nanofluids was observed to be lower than the base fluids [103]. The increase in particle concentration reduced the specific heat of the nanofluids. Low or high specific heat could be advantageous or disadvantageous depending on the engineering applications.

The suspension of nanoparticles in base fluids has been acknowledged to improve the thermo-physical properties of conventional fluids [104]. Nonetheless, due to the inconsistencies in the previous studies, the reason behind these improvements is still unclear. This section summarizes the experimental findings obtained from the previous research on the thermo-physical properties of nanofluids, including the thermal conductivity, dynamic viscosity, density, and specific heat of the hybrid nanofluids. Several factors have been reported to affect the thermo-physical properties of hybrid nanofluids, such as the type of nanoparticle, size, shape, base fluid, and operating temperature. These factors will be adequately addressed in the next section as reference and comparison to the current study.

### 4.1. Thermal Conductivity

Thermal conductivity (denoted as *k*) is one of the thermo-physical properties. According to Çengel and Ghajar [105], thermal conductivity is the measure of a material’s ability to conduct heat. For liquids, it is a measure of the ability to transfer heat. The thermal conductivity of liquids is located between solids and gases, where the highest thermal conductivity can be found in the solid phase and lowest in the gas phase. This property is important for the determination of the rate of heat transfer across materials. Nanofluids have been subjected to debate due to their high thermal conductivity characteristics. According to Sajid and Ali [69], the thermal conductivity is highly dependent on the concentration of nanoparticles, size, shape of nanoparticles, type of nanoparticle, temperature, and type of base fluid. The study on improving thermal conductivity is very important in the heat transfer process, as it influences the convective heat transfer of fluids [106,107]. The following section discusses in detail how these factors play a significant role in influencing the thermal conductivity of the nanofluids.

#### 4.1.1. Effect of Particle Concentrations

The addition of small amounts of nanoparticles can enhance the thermal conductivity of the conventional fluids significantly due to their high thermal conductivity [108]. Therefore, the thermal conductivity of nanofluids is expected to improve as the concentration of particles increases. Esfahani et al. [109] prepared the ZnO-Ag/water hybrid nanofluids at various volume fractions of 0.125 to 2.0%. The study reported that the effect of increasing the volume fractions is more significant than the temperature in the improvement of the thermal conductivity. Thermal conductivity was enhanced by up to 21.42% at 2.0% when compared to the 0.125% volume fractions. That is because the number of particles is higher at high volume fractions. These particles collide more frequently due to the Brownian motion effect, thus increasing the thermal conductivity. Sarbolookzadeh Harandi et al. [110] studied the effects of particle concentrations on the thermal conductivity of F-MWCNT-Fe_3_O_4_/EG hybrid nanofluids. The samples were prepared at concentrations between 0.0 and 2.3%. The maximum thermal conductivity enhancement was observed at a 2.3% volume fraction, which is 30%. The influence of particle concentration on thermal conductivity was observed to be more dominant at higher temperatures than at lower temperatures. In another study, Madhesh et al. [111] explained that the enhancement of thermal conductivity of nanofluids as volume concentrations increase is related to the development of closely packed thermal interfaces.

Zadkhast et al. [112] stated that the influence of temperature on the thermal conductivity enhancement was only evident at higher volume fractions. The highest percentage enhancement of thermal conductivity occurred at 0.6%. When the temperature was kept constant, the improvement in the thermal conductivity of MWCNT-CuO/water hybrid nanofluids varies from 9.61 to 30.38%. Similar trends were found in a study by Hemmat Esfe et al. [113] for the thermal conductivity of SiO_2_-MWCNT/EG hybrid nanofluids. The thermal conductivity of the hybrid nanofluids increased with the concentrations. By increasing the volume concentration from 0.05 to 1.95%, the enhancement in the thermal conductivity was observed to increase from 4.5 to 22.2%. However, there is no substantial enhancement in the thermal conductivity at concentrations less than 0.115%, and therefore is not suggested as a heat transfer fluid, particularly at low temperatures of 30 and 35 °C. In another study, Nabil et al. [64] measured the thermal conductivity of TiO_2_-SiO_2_/EG:W hybrid nanofluids at different volume concentrations ranging from 0.5 to 3.0%. The thermal conductivity ratio was found to be almost linear with the volume concentrations. In addition, Nabil et al. [64] also mentioned that the hybrid nanofluids at higher volume concentrations (*ϕ* ≥ 1.5%) were seen to behave as good heat transfer fluids based on the property enhancement ratio (PER).

Recently, green nanofluids with improved thermo-physical properties have received attention in nanofluids research due to their low toxicity characteristics. Khdher et al. [19] dispersed Al_2_O_3_/BG nanofluids for green base fluids of bio-glycol. The study on thermal conductivity was conducted at 0.1, 0.3, 0.5, 0.7, and 1.0% volume concentrations using KD2 Pro Thermal Property Analyzer. Based on the results, the thermal conductivity of nanofluids improved by up to 17% at volume concentrations of 1.0%. They explained that the enhancement in thermal conductivity was due to surface layering around nanoparticles that was formed by base fluid molecules, and these surface nanolayers have higher thermal conductivity than the base fluid [10]. Yarmand et al. [114] incorporated carbon that was synthesized from empty fruit bunches with graphene to produce a surfactant-free activated carbon-graphene oxide hybrid nanofluids (ACG/EG). They observed an increase in thermal conductivity with weight concentrations. Maximum thermal conductivity enhancement for the ACG/EG hybrid nanofluids is 6.47%, which can be found at a weight concentration of 0.06%. They further explained that the enhancement of thermal conductivity was the result of uniform dispersion of nanoparticles in the EG.

#### 4.1.2. Effect of Temperature

Most applications and systems that involve heating and cooling processes operate at a wide range of temperatures. Hence, many researchers manifested their study on the fluids suspended with nanoparticles at various operating temperature range, mostly from 5 to 80 °C. Prior records revealed that the thermal conductivity of nanofluid is highly dependent on temperature [76,84,102,115,116,117]. According to Lim et al. [102], the observed trend can be associated with the inclusion of nanoparticles in base fluids, since the measured base fluids did not show a notable increase in thermal conductivity when the temperature rose. In addition, published articles on temperature-dependent thermal fluids mentioned that these outcomes resulted from fierce Brownian motion at a high temperature, which is attributed to the enhancement of the nanofluids thermal conductivity [102,118,119].

Lim et al. [102] conducted an experimental study to measure the thermal conductivity of SiC/EG nanofluids at a temperature range between 20 and 50 °C. The results reveal that the thermal conductivity of SiC/EG nanofluids increases as temperatures increase, where the highest thermal conductivity was found at maximum temperature, 50 °C, augmented by up to 16.21% relative to the base fluids. Similarly, Mostafizur et al. [118] measured the thermal conductivity of Al_2_O_3_/methanol nanofluids at the low-temperature range, 5 to 25 °C. The maximum enhancement of thermal conductivity was reported to be 14.29% at a temperature of 25 °C. Aparna et al. [120] observed the increment in the thermal conductivity of aqueous Al_2_O_3_-Ag/water hybrid nanofluids with the temperature. The effect of temperature on the thermal conductivity was observed to be insignificant at lower particle loading; however, it was amplified at higher particle loading. This is because the number of nanoparticles is greater at higher particle loading. When the temperature increases, the Brownian motion caused the particle in the base fluids to collide more frequently at higher rates, which help the particles to transport heat faster, increasing the overall thermal conductivity [121]. However, Riahi et al. [122] stated that while the Brownian motion is the main mechanism that improves the thermal conductivity of nanofluids at low temperature, it is not the main effect that contributes the thermal conductivity enhancement at a higher temperature. Instead, it involves several other mechanisms such as layering, clustering, ballistic phonon motion, thermal boundary resistance, and mass difference scattering [123].

Riahi et al. [122] studied the effects of temperature on thermal conductivity of Al_2_O_3_/water nanofluids and found the thermal conductivity of nanofluids increased from 4.2% to 8.6% from the base fluid at a temperature range between 25 and 45 °C. They considered the Brownian motion as the primary reason behind this enhancement. In addition, convection that occurred due to the interaction between the solid nanoparticles and fluid molecules also contributed to the enhancement of the thermal conductivity with the temperature. In contrast, Shima et al. [124] reported a constant variation shown by the thermal conductivity ratio of iron oxide-kerosene, hexadecane, and water nanofluids with the temperature, revealing an insignificant effect of microconvection on the improvement of thermal conductivity. Sarbolookzadeh Harandi et al. [110] measured the thermal conductivity of F-MWCNTs-Fe_3_O_4_/EG hybrid nanofluids. The results show that the thermal conductivity of the hybrid nanofluids was enhanced from 19 to 30% within the temperature range of 25 to 50 °C. Megatif et al. [125] investigated TiO_2_-CNT/water hybrid nanofluids and observed a linear relationship between the thermal conductivity and temperature.

#### 4.1.3. Effect of Size and Shape

For years, one of the prominent ideas in improving heat transfer performance was by adding nanoparticles in conventional fluids [126]. The question that then naturally arises is how different types of nanoparticle affect the thermo-physical properties of nanofluids. Nanofluid can be classified into several categories, but three types of nanoparticle that are commonly used in this field of research are (i) carbon-based (e.g., CNTs, fullerenes), (ii) metal (e.g., Au, Ag, Cu, Fe), and (iii) non-metallic solids/ceramics (e.g., CuO, Al_2_O_3_, TiO_2_, SiO_2_) [127,128,129,130,131,132,133,134,135]. CNTs can be classified into SWCNT and MWCNT, and this type of nanoparticle demonstrates a considerably high thermal conductivity enhancement [136,137]. Since nanofluid thermal conductivity can be directly influenced by particle thermal conductivity [127], a proper selection of nanoparticles for the dispersion with base fluids may significantly enhance the thermal conductivity. Moldoveanu et al. [29] studied the thermal conductivity of three different water-based nanofluids, Al_2_O_3_, SiO_2_, and their hybrid at various concentrations. For the hybrid nanofluids, the Al_2_O_3_ and SiO_2_ were prepared at 1:1, 1:2, 1:3, and 1:5 mixture ratios. They found that SiO_2_/water nanofluids exhibited higher thermal conductivity than Al_2_O_3_/water nanofluids. However, the hybridization between Al_2_O_3_ and SiO_2_ nanoparticles resulted in lower thermal conductivity compared to SiO_2_/water nanofluids, but higher thermal conductivity compared to Al_2_O_3_/water nanofluids. The thermal conductivity for the hybrid nanofluids increased by up to 23.61% for Al_2_O_3_: SiO_2_ at a 1:5 mixture ratio. The results indicate that the addition of SiO_2_ nanoparticles at higher volume fractions produced higher thermal conductivity.

In another study, Moldoveanu et al. [138] conducted a similar investigation on thermal conductivity with Al_2_O_3_/water, TiO_2_/water, and Al_2_O_3_-TiO_2_/water hybrid nanofluids. The thermal conductivity of Al_2_O_3_/water nanofluids is lower than TiO_2_/water nanofluids at low volume fractions; however, as the volume fraction increases, the thermal conductivity of Al_2_O_3_ nanofluids rose higher than the TiO_2_/water nanofluids. On the other hand, the hybrid Al_2_O_3_-TiO_2_/water nanofluids demonstrated a higher thermal conductivity relative to the mono nanofluids. According to Sarkar et al. [100], the enhancement can be explained by the synergistic effects between Al_2_O_3_ and TiO_2_ nanoparticles. Smaller sized particles help in the conduction by filling in the spaces between larger particles and increase the thermal conductivity. Minea [139] estimated the relative thermal conductivity of Al_2_O_3_, TiO_2_, SiO_2_, Al_2_O_3_-TiO_2_, and Al_2_O_3_-SiO_2_ hybrid water-based nanofluids using different correlations. Interestingly, they found that the Al_2_O_3_/water nanofluid exhibited a higher relative thermal conductivity than the hybrid nanofluids, followed by Al_2_O_3_-TiO_2_/water hybrid nanofluids, and Al_2_O_3_-SiO_2_/water hybrid nanofluids. Pryazhnikov et al. [140] evaluated the thermal conductivity of different particle materials, namely ZrO_2_, Al_2_O_3_, TiO_2_, SiO_2_, and CuO, at the same particle size and concentration. The results infer that there is no direct correlation between relative thermal conductivity and thermal conductivity of the particle material. However, they found that the thermal conductivity was enhanced with particle material density.

Due to the poor thermal conductivity of conventional heat transfer fluids, constant improvement of the heat transfer fluid was made to increase the performance in the heating and cooling system. The addition of nano-scale particles in the base fluids undoubtedly enhanced the thermo-physical properties of the fluids. However, the factors contributing to the enhancement are still ambiguous and need a more in-depth investigation. Previous research observed several determinants that can be varied to influence the enhancement of thermal conductivity of nanofluids, including base fluids. The study on the effects of base fluids on thermal conductivity is important because one of the mechanisms behind the thermal conductivity enhancement of nanofluids, Brownian motion, is influenced by the viscosity of the base fluids, which influences the thermal conductivity [141]. Moreover, the improvement in thermal conductivity of nanofluids is also attributed to the thermal conductivity of the base fluids [106]. Common base fluids that were used in the suspension with nanoparticles are water, ethylene glycol, and propylene glycol. These fluids are easily available and comparable with the existing literature. So far, no definite trend has been reported on base fluid’s influence on the thermal conductivity of nanofluids as the thermal conductivity can be influenced by many other factors such as the type of nanoparticle, size and shape of particles, temperature, and particle concentration. However, its effect on thermal conductivity still needs to be considered.

As other variables such as temperature and particle loading were kept constant, the role of the base fluids in influencing the thermal conductivity of nanofluids is evident. The suspension of nanoparticles in initially high thermal conductivity base fluids usually results in high thermal conductivity of the nanofluids compared to nanofluids that were dispersed in base fluids with lower thermal conductivity. Idrus et al. [142] measured the thermal conductivity of carbon nanofibers (CNF) in different base fluids, deionized water (DI water) and ethylene glycol (EG), at 6, 25, and 40 °C using the KD-Pro Thermal Properties Analyzer. They found that the thermal conductivity of DI water-based nanofluids was enhanced by up to 39.6% from the base fluid, whereas the maximum thermal conductivity for EG-based nanofluids was 36.7%. DI water-based nanofluids demonstrated a higher thermal conductivity enhancement than EG-based nanofluids, which is attributed to the high thermal conductivity of DI-water. Similarly, [143] dispersed MWCNTs-OH nanoparticles in deionized water and ethylene glycol. When temperature and concentration were kept constant, the results from this study indicate that deionized water-based nanofluids demonstrated a higher thermal conductivity than ethylene-based nanofluids.

In another study, Akilu et al. [144] studied the thermal conductivity of ß-SiC in different base fluids, ethylene glycol (EG) and propylene glycol (PG). ß-SiC/EG nanofluids showed an outstanding increment in thermal conductivity from the base fluid compared to the ß-SiC/PG nanofluids. The maximum enhancement of thermal conductivity for EG-based nanofluids and PG-based nanofluids was 14.64% and 4.83%, respectively, at *T* = 60 °C and *ϕ* = 1.0 vol.%. The outcomes from the study are relevant to the thermal conductivity of the base fluids, where thermal conductivity of EG is higher than PG. Al-Waeli et al. [145] also performed an intensive investigation on the thermal conductivity of SiC nanoparticles dispersed in different base fluids; water (W), a mixture of water and ethylene glycol (W/EG), and a mixture of water and propylene glycol (W/PG). In the study, ethylene glycol and propylene glycol were mixed with water at a 35:65 mixture ratio. To properly study the effects of base fluids on thermal conductivity, they measured the thermal conductivity of W, W/EG, and W/PG based SiC nanofluids at low weight concentration (0.5 wt.%) and at a temperature range between 25 and 60 °C. The study observed an insignificant difference in the thermal conductivity enhancement for the SiC nanoparticles suspended in water, ethylene glycol, and propylene glycol. SiC nanofluids increased from 1.66 to 2.29% at temperatures between 25 and 60 °C relative to the base fluids. Khdher et al. [19] compared the thermal conductivity of Al_2_O_3_/BG green nanofluids with Al_2_O_3_/EG and Al_2_O_3_/PG nanofluids. The thermal conductivity of Al_2_O_3_/BG nanofluids was enhanced by up to 17%, while Al_2_O_3_/EG and Al_2_O_3_/PG nanofluids were enhanced by up to 9% and 3.6%, respectively at T = 30 °C.

Many of the studies performed by researchers demonstrated the interaction between the size of nanoparticles and the improvement of fluid thermal conductivity. Generally, nanofluids with a smaller particle size have been documented to provide a higher thermal conductivity enhancement relative to the larger particles [146,147]. Hemmat Esfe et al. [148] comprehensively studied the effects of particle size (37, 71, and 98 nm) on the thermal conductivity of Fe/water nanofluids. They found an increment in thermal conductivity of nanofluids when particle size was reduced, and the increment becomes more significant at higher volume concentrations. They explained that the nanofluids with smaller particle size have a greater surface area of the solid–liquid interface, which led to the enhancement in the thermal conductivity. The results of this research are consistent with prior research conducted by Chopkar et al. [149]. They investigated the effects of different particle sizes of Al_2_Cu/W:EG and Ag_2_Al/W:EG nanofluids on thermal conductivity. They reported that a higher thermal conductivity ratio was achieved with the particle size of 30 nm for both nanofluids. As the particle size increases, the thermal conductivity ratio decreases. They stated that other than the thermal conductivity, smaller particle size also improved the stability and homogeneity of the nanofluids. Similarly, Liu et al. [150] found that Al_2_O_3_/water nanofluids containing 30 nm particle size were first to show a separation layer after several days of observation, followed by the 20 nm particle size.

Teng et al. [151] also measured the thermal conductivity of Al_2_O_3_/water nanofluids at different concentrations, temperatures, and particle sizes of 20, 50, and 100 nm. When other variables were kept constant, the highest thermal conductivity ratio was found as being up to 14.7%, 7.3%, and 5.6%, respectively. The outcomes from these studies can be theoretically explained by using the concept of Brownian motion and liquids layering around particles [148]. This is due to the greater surface area of the solid–liquid interface found at smaller particle size that assists the thermal energy transfer. In addition, more vigorous Brownian motion obtained with smaller particle size also assisted the enhancement of thermal conductivity [127,148,152]. In another study, Hossein Karimi Darvanjooghi and Nasr Esfahany [153] prepared silica/ethanol nanofluids at various concentrations to investigate the effects of nanoparticle size (10.6, 20, 38.6, and 62 nm) on thermal conductivity. They found that the relative thermal conductivity increased when the particle size increased. At a volume fraction of 1.17%, a maximum of 70% enhancement in thermal conductivity was observed for nanofluids containing 62 nm sized particles. They clarified that the increment of relative thermal conductivity with particle size is related to the mitigation of interfacial thermal resistance. Although the size of nanoparticle seems to have an impact on the thermal conductivity of nanofluids, it is difficult to conclude whether it plays a significant role in the enhancement because of the difficulty in quantifying the immeasurable Brownian motion and surface effect [127].

In addition, the shape of particles is also one of the factors that need to be considered in the study related to the nanofluids. Studies on the effects of particle shape are very limited in the literature, and the correlation with thermal conductivity is hard to achieve as the different shapes of particles are diverse in size. Nevertheless, the discovery shown from prior investigations proves that the relationship between the shape of the particle and thermal conductivity exists. Murshed et al. [154] conducted an experimental study on the enhancement of thermal conductivity with spherical (D = 15 nm) and rod-shaped (10 nm × 40 nm) TiO_2_/water nanofluids. The authors reported that at a maximum of 5% volume concentration, both nanofluids augmented up to 30% and 33%, respectively. Similarly, Chen et al. [155] experimented with spherical TiO_2_ (25 nm) and rod-shaped TNT (10 nm × 100 nm) nanoparticles dispersed in water- and ethylene glycol (EG)-based fluids. The results divulge that for both water-based and EG-based nanofluids, the spherical-shaped nanoparticles show a slightly higher increment of thermal conductivity and significantly lower viscosity compared to the rod-shaped nanoparticles. Jeong et al. [156] compared the thermal conductivity of the sphere and rectangular shape of ZnO/water nanofluids. The results reveal that the thermal conductivity of rectangular-shaped ZnO/water nanofluids is higher than sphere-shaped ZnO/water nanofluids. By increasing the volume concentration from 0.5 to 5.0%, rectangular-shaped ZnO/water nanofluids showed an increase in thermal conductivity from 3.0 to 19.8%, while a 2.5 to 16.0% increase was observed for the sphere-shaped ZnO/water nanofluids.

In another study, Timofeeva et al. [157] investigated the effects of particle shape on the thermo-physical properties of alumina/EG:W nanofluids. The results obtained from the experiment suggest similar outcomes to the studies mentioned earlier. Four different shapes of alumina nanoparticles, namely platelets (9 nm), blades, cylinders (80 × 10 nm), and bricks (40 nm), were dispersed in a mixture of ethylene glycol and water at different volume concentration. The results demonstrate that the enhancement of thermal conductivity at room temperature according to the shape of particles is cylinders > bricks > blades ≈ platelets. According to Sezer et al. [46], nanofluids with cylindrical shape particles demonstrated a higher thermal conductivity than spherical-shaped particles due to the high aspect ratio. Nanoparticles with higher aspect ratio are capable of transporting heat faster over a considerable distance. Ghosh et al. [158] stated that the thermal conductivity enhancement is related to the collision between nanoparticles and heat source. Thus, they performed a molecular dynamic simulation for the spherical-shaped Cu nanoparticles and cylindrical-shaped Cu nanoparticles with aspect ratio of 2 and 4. Based on the temperature variation, the cylindrical nanoparticles with an aspect ratio of 4 collected thermal energy more rapidly during the collision with the heat source than the cylindrical nanoparticles with an aspect ratio of 2 and spherical nanoparticles. Farbod et al. [159] investigated the thermal properties dependence on the morphology by using CuO/oil nanofluids for different nanostructure. Nanofluids with nanorod structure demonstrated the highest thermal conductivity increment of 8.3% relative to the containing nanoparticles and nanorhombic structure. Together, the findings confirm a relationship between particle shape and thermal conductivity of nanofluids. However, it remains unclear to what degree particle shape is attributed to the thermo-physical properties of the nanofluids. A summary of thermal conductivity enhancement of various nanofluids is tabulated in Table 4.

### 4.2. Dynamic Viscosity

In nanofluids research, the interest in formulating thermal fluids with high thermal conductivity arose centuries ago. Even so, having high thermal conductivity is not the only condition a liquid should possess for it to be employed as a coolant effectively. Its viscosity also needs to be considered [162]. Çengel and Ghajar [105] defined viscosity as a measure of the fluid resistance to gradual deformation by shear stress. Viscosity is one of the temperature-dependent fluid properties that are as important as thermal conductivity. While nanoparticle suspended fluids have shown a significantly higher viscosity relative to their base fluids, most nanofluids show exceptional heat transfer [163]. Viscosity is another property that determines the fluid resistance to flow in a system. Since it plays a significant role in determining the energy required for the fluids to flow, it becomes one of the major focus areas in many research fields. In other words, high viscosity will result in more pumping power [164], and thus increased energy usage. This statement is aligned with the research made by Torii [165]. They disclosed that the pumping power required to pump Al_2_O_3_, CuO, and diamond nanofluids at 5% volume concentration at the same velocity is higher compared to the base fluids.

Viscosity is expected to be higher than its base fluid, contributing to higher pumping power and a lower heat transfer coefficient [162]. Despite experiencing an increase in viscosity when nano-scaled particles were added to the base fluid [166], enhancement of heat transfer displayed by this new engineered fluid is considered outstanding compared to conventional fluid [163]. Using high viscosity fluid to transport heat may cause an increase in pressure drop; however, nanofluids are anticipated to display a high thermal conductivity without increasing the pressure drop [44]. Unlike thermal conductivity, the viscosity of thermal fluids exponentially decreases with temperature and increases with concentration. It is commonly known, that viscosity is temperature-dependent and can change with an increase and decrease in fluid concentration. However, other vital factors such as base fluids, type of particle, shape, and size of particles also need to be considered in the preparation of nanofluids [163,167]. Although similar nanofluids were used to study viscosity, the results may vary between each study. Previously, Duangthongsuk and Wongwises [168] compared the viscosity of TiO_2_/water nanofluids with other researchers and found discord in the data. This may be caused by the nanofluids preparation method, size of nanoparticles, measurements techniques used, and nanoparticles’ method of preparation. The investigation performed by Duangthongsuk and Wongwises [168] showed that the viscosity of TiO_2_/water is about 4–15% higher than its base fluids.

#### 4.2.1. Effect of Particle Concentration and Temperature

Yu et al. [169] performed an investigation to study the viscosity of ZnO/EG nanofluids at 0.02 to 0.05% volume concentrations. The rheological behavior of ZnO/EG nanofluids was evaluated at a temperature of 20 to 60 °C. From the study, the viscosity of ZnO/EG nanofluids was found to decrease as temperature increases. The minimum viscosity was found at a concentration of 0.02% and a temperature of 60 °C. Authors also revealed that at low concentrations, ZnO/EG nanofluids possessed Newtonian behavior. Sundar et al. [170] observed declining trends in viscosity as the temperature increased for SiO_2_/water and ZnO/water nanofluids that were measured in the temperature range from 20 to 80 °C. In another study, Azmi et al. [76] investigated the effects of temperature on TiO_2_/W:EG nanofluids and found that the viscosity decreased with increasing temperature but increases with concentration. The viscosity of TiO_2_/W:EG nanofluids was found to be maximized at 1.5% volume concentration and a temperature of 30 °C. A fluctuation in relative viscosity was observed at a working temperature of 30 to 80 °C. They explained that the fluctuation in the relative viscosity may be due to the dissimilar structure and different diffused layer thickness around the surface of nanoparticles.

Researchers have also conducted numerous experimental investigations to study the viscosity of the hybrid nanofluids. Nabil et al. [64] investigated the viscosity of TiO_2_-SiO _2_/W:EG hybrid nanofluids at various concentrations and temperature. The viscosity of the hybrid nanofluids was observed to be higher than the base fluid, W/EG, at all concentrations. This is because the dispersion of nanoparticles in the base fluid increases the internal shear stress, resulting in increased viscosity. They found the maximum increase in viscosity of the hybrid nanofluids, 62.5%, at 3.0% volume concentration. Nevertheless, the viscosity trends shown by the hybrid nanofluids follow the base fluid’s behavior, which decreases with temperature. The rheological behavior of G-SiO_2_/water hybrid nanofluids at different solid volume concentrations and temperatures was investigated by Kazemi et al. [73]. They found that the base fluids showed Newtonian behavior, while G-SiO_2_/water hybrid nanofluids showed non-Newtonian behavior. At a constant shear rate, the viscosity of the hybrid nanofluids increases with the particle loading and decreases with temperature. They stated that the increase in internal friction due to the addition of nanoparticles increases the flow resistance, and results in a higher viscosity. The viscosity of the hybrid nanofluids in the study experienced an increase by up to 173% from the base fluids at 1.0% volume concentration. On the other hand, the increase in temperature undermines the bond between the base fluid’s molecules and particles. Aside from this, the van der Waals forces between particles decreased at high temperatures and mitigated the movement of the fluid layers on each other. Consequently, the viscosity of the hybrid nanofluids is reduced.

Other than thermal conductivity, researchers have also actively studied the viscosity of eco-friendly nanofluids. Adewumi et al. [171] explored the effects of temperature and mass fractions on the viscosity of W/EG-based green nanofluids containing carbon nanoparticles of coconut fiber. The study reported an increase in the viscosity with mass fraction and decreased with temperature. However, they reported no significant increase in relative viscosity with the temperature. The highest viscosity was observed at a mass fraction of 1.0%, which was double that of the base fluids. Sadri et al. [10] evaluated the viscosity of green rGO/water nanofluids at various concentrations ranging between 0.02 and 0.08%. The viscosity of the nanofluids decreases as the temperature increases, which follows the trend exhibited by the base fluids. From the study, an insignificant increase was observed for the viscosity of nanofluids with particle concentrations. They stated that good dispersion of nanoparticles with the least agglomeration reduced the particle interference in the shear-strain fluid system, thereby minimizing the increase in viscosity from the base fluids. Meanwhile, Yarmand et al. [114] synthesized carbon from waste materials to produce a hybrid nanofluids with graphene in ethylene glycol at various weight concentrations. They reported a minimal increase in the viscosity of hybrid nanofluids with the weight concentrations. The viscosity increased non-linearly with weight concentrations, and this was due to the increase in liquid internal shear stress.

Similarly, Abdolbaqi et al. [21] investigated the effects of volume concentrations and temperature on TiO_2_/W:BG green based nanofluids at 0.5 to 2.0% volume concentrations and temperature of 30 to 80 °C. The viscosity of TiO_2_/W:BG nanofluids was observed to be higher than the base fluids and increased with volume concentration, whereas the increase in temperature reduces the viscosity of the green nanofluids. At 2.0% volume concentration, nanofluids viscosity increased from 20.5 to 33.8% and 29.8 to 53.4%, respectively, for the mixture of W/BG at 80:20 and 70:30 in the range of 30 to 80 °C. In another study, Abdolbaqi et al. [22] also found a similar trend for the viscosity of SiO_2_/W:BG-based green nanofluids with concentration and temperature. For the mixture of W/BG at 80:20 and 70:30, the viscosity of SiO_2_/W:BG nanofluids increased from 16.02 to 28.9% and 17.3 to 37.8%, in the temperature range of 30 to 70 °C and 30 to 60 °C, respectively. In comparison to TiO_2_/W:BG nanofluids ([21], SiO_2_/W:BG nanofluids demonstrated a lower viscosity increment at similar conditions. According to Kazemi et al. [73], the minimum increment of viscosity for nanofluids with SiO_2_ nanoparticles is attributed to its spherical shape and low specific surface area that enables the base fluid layers to slide freely on each other.

#### 4.2.2. Effect of Size and Shape

Nikkam et al. [162] measured the viscosity of *α*-SiC/W:EG and *β*-SiC/W:EG nanofluids with different nanoparticles shapes—hexagonal and almost-spherical shape, respectively—at a temperature of 20 °C. From the study, the viscosity of *β*-SiC/W:EG nanofluids was observed to be higher than the *α*-SiC nanofluids. They mentioned that the possible explanation behind this trend was due to the difference in surface area between *α*-SiC/W:EG and *ß*-SiC/W:EG nanoparticles. The *α*-SiC/W:EG nanoparticles have a much smaller surface area (18 mm) than *ß*-SiC/W:EG (80 and 90 mm), which reduces the contact area between particles and the base fluid, which led to a reduction in the viscosity. In another study, Azevedo Oliveira et al. [172] dispersed different sizes of silver nanoparticles (10 and 80 nm) in water to study their effects on thermo-physical properties of the nanofluids. From the results, the viscosity of Ag/water nanofluids with nanoparticle size of 10 nm was found to be higher than the Ag/water nanofluids containing nanoparticles with a size of 80 nm for volume concentrations of more than 0.2%. The highest relative viscosity was found at 0.2% volume concentrations, for nanofluids containing 10 nm sized silver nanoparticles. A prior experimental study conducted by Namburu et al. [173] on the viscosity of SiO_2_/EG:W nanofluids containing different sized nanoparticles (20, 50, and 100 nm) also proves that the size of nanoparticles is capable of altering the physical properties of nanofluids. However, in contrast with the trend presented by Nikkam et al. [162], they reported that higher viscosity was observed at smaller particle sizes. More recent research conducted by Timofeeva et al. [174] showed consistency with previously discussed results where viscosity is much lower at a bigger size of particle than smaller particle size because at larger particles, the solid and liquid interfacial area is smaller.

Nithiyanantham et al. [175] studied the effects of different shapes of Al_2_O_3_ nanoparticles in eutectic salt on the viscosity at various temperatures. They found that the suspension of 13 nm spherical-shaped and 50 nm nanorod-shaped Al_2_O_3_ nanoparticles in the base fluids at 1.0 wt.% caused an increase in the viscosity. In contrast, the viscosity of the nanofluids was observed to reduce when temperature increases. This is because intermolecular attractions are weakened at a higher temperature [176]. Relative to the base fluids, the viscosity of spherical-shaped and nanorod-shaped Al_2_O_3_/eutectic salt nanofluids increased from 5 to 25% and 12 to 37%, respectively, in the temperature range between 250 and 400 °C. Timofeeva et al. [157] prepared Al_2_O_3_/W:EG nanofluids. They investigated the effect of particle shape on the thermo-physical properties of Al_2_O_3_/W:EG nanofluids at various volume concentrations and temperatures. The study revealed that the viscosity of nanofluids was dependent on the particle shape, and the differences in viscosity for each particle shape becomes apparent when the concentration increases. The viscosity for platelet-shaped Al_2_O_3_/W:EG nanofluids was more than two times higher than the blade-shaped Al_2_O_3_/W:EG nanofluids. The ascending sequence in the viscosity of Al_2_O_3_/W:EG nanofluids according to shape is blades < bricks < cylinders (rods) < platelets. They also modified the pH of the cylinder-shaped Al_2_O_3_/W:EG nanofluids to 2.54, 3.33, and 4.10 by adding 2N HNO_3_. The results show that the viscosity of the nanofluids decreased when pH value decreased, without any significant changes in the enhancement of thermal conductivity.

The dispersion of nanoparticles with different types of base fluid could give different outcomes in the viscosity measurement. This statement can be supported by the investigation undertaken by Abdullah et al. [143], which investigated the viscosity of MWCNT-OH dispersed in two different base fluids, deionized water and ethylene glycol, at various concentrations ranging from 0.1 to 1.0 wt% at various temperatures. Despite using the same type of nanoparticles, the highest viscosity of MWCNT-OH nanofluids was found at different concentrations, which is 0.6 wt% for deionized water at 40 °C and 1.0 wt% for ethylene glycol-based fluids at 6 °C. They reported an irregular pattern in viscosity of MWCNT-OH/water nanofluids, which is that at 6 °C, the viscosity of nanofluids at 0.4, 0.8, and 0.9 wt% is lower than the base fluid. The study observed the fluctuation in viscosity and did not follow the trend shown by the base fluids. Unlike MWCNT-OH/water nanofluids, MWCNT-OH/EG nanofluids showed an expected result and followed the base fluids in terms of its viscosity decreasing as the temperature increases. Lim et al. [102] observed the effects of base fluids on the viscosity of Al_2_O_3_/W:EG nanofluids. In the study, the suspension of Al_2_O_3_ nanoparticles at various concentrations in 60:40, 50:50, and 40:60 of W:EG increased the viscosity of the nanofluids. When temperature and particle concentrations were kept constant, the increase in viscosity was 1.39, 1.35, and 1.23 times for mixture of W/EG at 40:60, 60:40, and 50:50. The results indicate that higher EG content results in a higher viscosity of nanofluids. A summary of dynamic viscosity behavior for various nanofluids is tabulated in Table 5.

### 4.3. Density

Various aspects, including density, may influence the efficiency of the heat transfer. However, compared to the thermal conductivity and dynamic viscosity, the study on density is very limited in the literature. Other than viscosity, fluid density is also a significant parameter in determining pressure drop. High viscosity and density will result in a high pressure drop, which is not favorable in a heat transfer application [182]. Hence, several investigations related to the density of nanofluids are reviewed in this section. According to Al-Waeli et al. [145], the power needed to rotate the pump increases when the density of the working fluids increases. They investigated the effects of the base fluid mixture on the density and found a decreasing trend for density when the temperature increases. They also compared the density of different base fluids with the addition of SiC nanoparticles. The results indicate that the dispersion of nanoparticles in the base fluid increases the density to a higher level than the base fluid. Between the dispersion of SiC nanoparticles in water, the mixture of water and ethylene glycol (65:35), and the mixture of water and propylene glycol (65:35), the highest density increase was observed in SiC/W:EG nanofluid. These outcomes supported the statement made by Teng and Hung [103] in their study. They have noted that the increase in density when adding nanoparticles in the base fluid is only natural because the density of nanoparticles is usually higher than the base fluid itself.

Chavan and Pise [183] measured the density of the different types of oxide nanoparticles in the base fluids using the Anton Paar DMA 500 Density Meter. The measurement of density was undertaken at volume concentrations between 0.1 and 1.0% and a temperature of 30 °C. Based on the results, the relative density of TiO_2_/water and Al_2_O_3_/water nanofluids were found to increase with the volume concentrations linearly. These nanofluids also demonstrated a higher relative viscosity than water based and EG based SiO_2_ nanofluids. The maximum relative viscosity for water-based Al_2_O_3_, TiO_2_, SiO_2_ nanofluids and EG-based SiO_2_ nanofluids at 1.0% volume concentration is 2.6%, 2.6%, 1.25%, and 0.954%, respectively. The results also indicate that the density differs with different base fluids. The SiO_2_/EG nanofluids yield much lower density increments than the SiO_2_/water nanofluids at the same concentration. They also reported an increase in the relative density with the increase in particle concentrations; however, a negligible decrease was observed in the relative density of Al_2_O_3_/water nanofluids with temperature. The estimation of density for Al_2_O_3_/water nanofluids was also conducted using a mixture ratio developed by Pak and Cho [184]. They found that there is accuracy between the experimental and estimated data with 0.08% maximum deviation. The density of the nanofluids estimated using the mixture ratio, as expressed in Equation (2), was developed based on mass balance analogy. Pak and Cho [184] used ℽ-Al_2_O_3_ and TiO_2_ nanoparticles in their study to investigate the heat transfer of the dispersed water. They used Equation (2) to estimate the density of the colloidal suspension. They stated that the maximum deviation between experimental and estimated density was spotted at a volume concentration of 0.6%, which is 31.6%. The findings from these studies confirm the reliability of the mixture ratio equation in estimating the density of nanofluids.

(2) $$\rho_{nf}=(1-\varphi )\rho_{bf}+\varphi_{nf}\rho_{p},$$

where $\rho_{nf}$, $\rho_{bf}$, $\rho_{p}$, and φ represent the density of nanofluids, density of base fluids, density of nanoparticles, and volume fraction of nanofluids, respectively.

In another study, Lim et al. [102] estimated the density of SiC/W:EG nanofluids at concentrations between 0 and 1.0% using Equation (3). From the investigation, the density of nanofluids increased with volume fraction. Equation (3) successfully estimated the density of SiC/W:EG nanofluids with a relatively small deviation. Meanwhile, Kishore et al. [185] used an equation proposed by Takabi and Salehi [101] that was developed based on Equation (2) to estimate the density hybrid nanofluids. The mixture relation equation to estimate the density of hybrid nanofluids is given by Equation (3). In the study, the 30:70 and 70:30 particle ratios of Cu-GnP/water hybrid nanofluids were prepared using the two-step method. They reported an increase in the density of hybrid nanofluids with volume concentrations. On the contrary, the density reduces as the temperature increases. Interestingly, the density of 70:30 Cu-GnP/water hybrid nanofluids was higher than the 30:70 particle ratio. However, these outcomes are reasonable since the density of Cu nanoparticles is initially greater than that of GnP nanoparticles. The results indicate that the density of hybrid nanofluids also depends on the type of nanoparticles and their mixing ratio. The maximum increase in density for 70:30 Cu-GnP/water hybrid nanofluids was found to be at 0.02% volume concentration with 12.5%.

(3) $$\rho_{hnf}=(1-\varphi )\rho_{bf}+\varphi_{p1}\rho_{p1}+\varphi_{p2}\rho_{p2},$$

where $\rho_{hnf}$, $\rho_{p1}$, and $\rho_{p2}$ represent the density of hybrid nanofluids, the density of nanoparticle type 1, and the density of nanoparticle type 2, respectively.

Sadri et al. [186] dispersed functionalized graphene with gallic acid (GAGNPs) using a non-corrosive and eco-friendly covalent method. The functionalized GAGNPs were dispersed in distilled water at 0.5% volume concentration. From the study, the density of GAGNPs/water nanofluids insignificantly decreased with temperature. They explained that the trend observed for the density of nanofluids is due to the thermal expansion at the higher temperatures. When the temperature increased from 20 to 40 °C, the density decreased by 0.6%. In another study, Sadri et al. [187] conducted the density measurement for water-based clove-treated graphene nanoplatelets (CGNPs) using the Mettler Toledo DE 40 density meter. The measurement was performed withing a range of temperatures between 20 and 40 °C for concentrations of 0.025 to 0.1 wt.%. They found a negligible increase in the density of nanofluids with the concentration of the particles. However, the decrease in the density was found to be approximately 6% in the range of 20 to 40 °C. Similarly, Sadri et al. [10] used the Mettler Toledo DE 40 density meter to evaluate the density of clove-treated MWCNTs (C-MWCNTs) at temperatures between 20 and 40 °C. The C-MWCNTs was dispersed in distilled water at 0.075, 0.125, and 0.175 wt.%. A maximum increase of 0.08% in the density was observed for C-MWCNTs/water nanofluids at 0.175 wt.%. They explained that the addition of C-MWCNTs in water increases the density because the density of C-MWCNTs itself is higher than that of water. At the same particle concentration, the density of nanofluids was reduced by 0.6% as temperatures increased from 20 to 40 °C.

Sharifpur et al. [188] studied the effect of nanolayer on the density of nanofluids. They measured the density for SiO_2_/water, MgO/glycerol, CuO/glycerol, and SiOx/EG:W nanofluids for volume concentration of 1 to 6% and temperature of 10 to 40 °C. They found that the density evaluation by the mixture model from the literature was predicted to have a higher value than the density from the experimental data. The deviation of the data was increased with increase in volume concentration. They concluded that the limitation of the existing mixing model is due to the nanolayer density between void and the base fluid density. Sharifpur et al. [188] developed a new model to predict the density of nanofluids with the addition of the equivalent nanolayer void thickness. However, they stated that the existing mixture model still can predict the density of nanofluids for volume concentration lower than 1%.

### 4.4. Specific Heat

Specific heat can be defined as the heat that is required to increase the temperature of the unit mass of a substance by one degree [189]. It is one of the essential properties in heat transfer, other than thermal conductivity and dynamic viscosity. Specific heat can be associated with thermal conductivity. The relationship between specific heat and thermal conductivity is presented in Equation (4).

(4) $$k=\alpha \rho C_{p},$$

where *k* is the thermal conductivity, *α* is the thermal diffusivity, *ρ* is the density, and *C_p_* is the specific heat.

Depending on the application, high specific heat could be advantageous or disadvantageous to the system. In a cooling system for a car engine, a high specific heat fluid is beneficial as it can absorb a large amount of heat without a significant rise in temperature, whereas in the applications that need a rapid change in temperature, fluid with low specific heat would be advantageous to the system. Previous investigations have shown a decrease in specific heat with the addition of nanoparticles in the base fluid. Teng and Hung [103] reported that the act of dispersing nanoparticles in the base fluid would decrease the specific heat of nanofluids. The reason behind this behavior was previously explained by He et al. [190]. They listed two crucial factors that affect the specific heat of the nanofluid, which are the change in interfacial free-energy of solid–liquid when nanoparticles are added into the base fluid, and the specific heat of nanoparticles and its base fluid. Nanoparticles with lower specific heat than the base fluid can lead to a decrease in the specific heat of the fluid after the suspension. On the other hand, the specific heat of the nanofluids will increase if the nanoparticles used higher specific heat than the base fluid.

Due to the limited experimental data on the specific heat of nanofluids, many researchers employed the existing models to predict the specific heat of nanofluids. The first model is a correlation that was developed by Pak and Cho [184] to estimate the specific heat of nanofluids based on the volume fraction, and expressed by Equation (5).

(5) $$c_{p.nf}=\left(1-\varphi \right)c_{p,bf}+\varphi c_{p,np},$$

where $C_{p,nf}$, $C_{p,bf}$, and $C_{p,np}$ represent the specific heat of nanofluids, the specific heat of the base fluid, and the specific heat of nanoparticles, respectively.

Another model that is commonly used in specific heat is given by Equation (6). Equation (6) was developed by Buongiorno [191] with the assumption that the base fluid and the nanoparticles are in a thermal equilibrium condition.

(6) $$c_{p,nf}=\frac{\left[\left(1-\varphi \right){\left(\rho c_{p}\right)}_{bf}+\varphi {\left(\rho c_{p}\right)}_{np}\right]}{\left[\varphi \rho_{np}+(1-\varphi )\rho_{bf}\right]},$$

where $C_{p,nf}$, $C_{p,bf}$, and $C_{p,np}$ represent the specific heat of nanofluids, the specific heat of base fluid, and specific heat of nanoparticles, respectively. Meanwhile, $\rho_{bf}$ and $\rho_{np}$ represent the density of the base fluid and density of nanoparticles, respectively.

The estimation of the specific heat of hybrid nanofluids is presented in Equation (7). $C_{hnf}$, $C_{p,bf}$, $C_{p,p1}$, and $C_{p,p2}$ represent the specific heat of hybrid nanofluids, the specific heat of base fluid, the specific heat of nanoparticle type 1, and the specific heat of nanoparticle type 2, respectively. Meanwhile, $\rho_{bf}$, $\rho_{p1}$, $\rho_{p2}$, and $\rho_{hnf}$ represent the density of the base fluid, the density of nanoparticle type 1, the density of nanoparticle type 2, and the density of hybrid nanofluids, respectively. Equation (7) was derived based on the correlation developed by Pak and Cho [184] and was initially employed in an experiment conducted by Takabi and Salehi [101].

(7) $$C_{hnf}=\frac{(1-\varphi ){\left(\rho C_{p}\right)}_{bf}+\varphi {\left(\rho C_{p}\right)}_{p1}+\varphi {\left(\rho C_{p}\right)}_{p2}}{\rho_{hnf}},$$

Kulkarni et al. [192] set up an experiment to measure the specific heat of Al_2_O_3_/EG:W nanofluids at various particle concentrations. The experimental data were compared using Equations (5) and (6). They reported that the specific heat estimated using Equation (6) showed better accuracy with the experimental data than the specific heat estimated by Equation (5). In another study, Zhou et al. [193] noted that in order to determine changes in the temperature of nanoparticles and fluids, the specific heat capacity plays an important role. The temperature changes in nanofluids will influence the temperature field of the nanofluids and affect the heat transfer and flow status. In their study, the specific heat of the CuO/EG nanofluids was measured at concentrations between 0.1 and 0.6%. They found that the specific heat of CuO/EG nanofluids decreased when the volume concentration of nanoparticles increased. The experimental data for specific heat were then compared using Equations (5) and (6). They reported a good agreement between the thermal equilibrium model and experimental data.

On the other hand, an investigation performed by Shin and Banerjee [194] reported that the thermal equilibrium model (Equation (6)) underestimated the specific heat of SiO_2_/chloride eutectic nanofluids at a weight concentration of 1%. They also reported that the specific heat of nanofluids was almost constant in the range of temperatures between 500 and 555 °C. Duangthongsuk and Wongwises [195] measured the specific heat of Al_2_O_3_-MEMPCM/water hybrid nanofluids using a differential scanning calorimeter. The measurement was conducted at various particle concentrations and temperature of 30 °C. Based on the findings, the specific heat of Al_2_O_3_-MEMPCM/water hybrid nanofluids was observed to be lower than the base fluid and decreases when volume fraction increases. They also reported that Equation (7) successfully predicted the specific heat of the hybrid nanofluids. Hamid et al. [78] and Madhesh et al. [111] also employed Equation (7) in their study to estimate the specific heat of TiO_2_-SiO_2_/W:EG and water-based Cu-TiO_2_/W:EG hybrid nanofluids, respectively.

In recent research, Sadri et al. [187] investigated the thermo-physical properties of stabilized clove-treated graphene nanoplatelets (CGNPs) in the water at various particle concentrations and temperature. The results from the study indicate that the specific heat of CGNPs/water nanofluids was lower than the base fluid. By increasing the concentrations from 0.025 to 0.1 wt%, the average decrease in the specific heat of CGNPs/water nanofluids from the base fluid was 0.43 to 1.52%. It is very clear from the result that the decrease in specific heat when particle concentrations increased was insignificant, whereas a slight increase in the specific heat of nanofluids was observed with the increase in temperature. Meanwhile, a study conducted by Kumaresan and Velraj [196] found opposite outcomes from the research mentioned above for the MWCNT/W:EG nanofluids. Ironically, they reported that the specific heat of MWCNT/W:EG nanofluids was enhanced from the base fluid at all volume fractions. They explained that this behavior could be due to the high surface area of the MWCNT nanoparticles per unit volume, resulting in high surface energy. Another interesting finding from the study was that the maximum specific heat was found at the lowest volume concentration (0.15%), and it was further decreased when the volume fraction increased.

## 5. Conclusions

A comprehensive review of nanofluids has been presented based on previous findings concerning current developments on green nanofluids, preparation stability, and thermo-physical properties of nanofluids. Two types of methods used in nanofluid preparations are the one-step method and two-step method. Several factors need to be considered in the selection of preparation methods, such as the type of nanoparticle and production size. The one-step method eliminates several steps involved in the two-step method, including storing, drying, dispersing, stirring, and sonicating. Eliminating these processes helps to reduce the agglomeration of nanoparticles in the base fluid and results in more stable nanofluids. However, this method is not suitable for large-scale production of nanofluids. The two-step method is more preferable as an economical method to prepare nanofluids.

Nowadays, nanoparticles are commercially produced in powder or liquid form and can be easily obtained. Despite this, nanofluids prepared using this method might face stability issues, where nanoparticles agglomerate before they are completely dispersed in the base fluid. Stability is one of the key factors in the production of nanofluids, as it can influence the thermal properties of nanofluids. Hence, to overcome this problem and achieve a stable suspension, several techniques were employed to enhance the stability of nanofluids prepared using the two-step method, such as ultrasonic agitation and pH adjustment. The efficacy of these techniques may vary according to the type of nanoparticles, type of base fluids, nanoparticle concentrations, and sonication time. However, several investigations reviewed in the literature showed that some nanofluids showed long-term stability without the use of surfactants and pH adjustments. Then, the stability of the nanofluids can be evaluated using several methods such as visual observation, transmission electron microscopy (TEM), and ultraviolet-visible spectroscopy.

The addition of mono nanoparticles in the base fluids led to an anomalous increase in the thermal conductivity compared to the conventional base fluids. This finding led to the formulation of hybrid nanofluids, where two or more dissimilar nanoparticles were dispersed in the conventional fluids. Based on the available literature, to fully employ the nanofluids as the new heat transfer fluid, a specific condition that gives optimum heat transfer performance needs to be investigated. Although the literature reported a discrepancy in the mechanisms that increase or decrease the thermo-physical properties of nanofluids, many types of research displayed their dependence on the nanoparticle concentrations, size and shape of nanoparticles, type of nanoparticle, operating temperature, and the type of base fluid. These variables are known to positively or negatively affect the thermo-physical properties of nanofluids. The addition of nanoparticles in the base fluids enhanced the thermal conductivity of the nanofluids.

As the evaluation of nanofluids advances from mono to hybrid nanofluids, there is concern among researchers about the adverse effects of nanofluids on humans and the environment. There have been several prior studies that designed eco-friendly methods to prepare nanofluids by synthesizing nanoparticles from various natural resources. The literature reported many successful formulations of nanofluids using these eco-friendly methods of nanoparticle synthesis in improving thermal conductivity and convective heat transfer with a minimum increment of friction factor. However, various researchers performed the forced-convection heat transfer experiment using oxide nanoparticles in water, ethylene glycol, or a mixture of water and ethylene glycol. The study of hybrid oxide nanoparticles in the green base fluid is underexplored in the literature.

## Figures and Tables

**Table 1:** Summary preparation of nanofluids.

Author(s)	Nanoparticles/Base Fluid	Preparation Method
[24]	CuO/EG	One-step method (Chemical reduction method)
[25]	Di-Ag/EG	One-step method (Polyol method)
[26]	TiO_2_-CuO and C/EG	Two-step method
[27]	Cu/EG and DEG	Two-step method
[28]	SiO_2_-Graphite/Water	Two-step method
[29]	Al_2_O_3_-SiO_2_/Water	Two-step method
[30]	Si_3_N_4_/EG	Two-step method
[31]	Al_2_O_3_-TiO_2_/NA	Two-step method
[32]	TiO_2_/Water	Two-step method
[18]	Si/Water	Two-step method
[33]	SWCNT- MgO/EG	Two-step method
[34]	Ag and Au/Water	One-step method
[35]	Cu/Methanol	Two-step method
[36]	CuO-TiO_2_/Water	Two-step method

**Table 2:** Stability behavior of nanofluids [88].

Zeta Potential (mV)	Stability Behavior
<±5	Rapid coagulation
±10 to ±30	Incipient stability
±30 to ±40	Moderate stability
±40 to ±60	Good stability
>±61	Excellent stability

**Table 3:** Summary of stability improvement and evaluation methods.

Author(s)	Nanofluids	Enhancement Method (s)	Evaluation Method (s)	Remarks
[92]	CuO/W	Ultrasonication: 1–4 h	-	Thermal conductivity increases with sonication time, temperature, and amount of surfactant.
	Al_2_O_3_/W	SDBS		
[83]	TiO_2_/W	Ultrasonication: 1.5 h	TEM	Raw TiO_2_ powder: agglomerated synthesized TiO_2_: smaller size and spherical shape.
		PVP Tween 20		
[85]	Al_2_O_3_/EG	Ultrasonication: 12 h	SEM UV-Vis	There is no distinguishing difference between the absorbance of the sample that was measured on the first day and fifth day.
		pH: 2–11		
		PVP SDBS		
		pH: 2–12		
	Al_2_O_3_/W	Anionic SDBS		
[93]	ND-Ni/W	Ultrasonication: 3 h	XRD DLS TEM SEM	The size of particles was also estimated using DLS: ND: 14 nm; Ni: 11 nm; ND-Ni: 28 nm - SEM: The suspension of ND-Ni particles in water in a quasi-spherical shape.
		Nanosperse AQ		
[94]	TiO_2_-MWCNT/W-EG	Ultrasonication: 45 min	Visual Observation DLS TEM SEM	- TiO_2_ water-based nanofluids were stable at pH = 9. - Sample nanofluids with no pH modification and less surfactant addition displayed better stability. - After 72 h observation of the sample, nanofluids with low surfactant quantity offered better stability.
		pH: 3, 6, 9, 12		
		CTAB		
[95]	CNT-SiO_2_/W	Ultrasonication: 3 h	SEM	- Sample at 2% vol. fractions: stable for up to 120 h after adding surfactant - SEM images showed CNT and SiO_2_ clustered and moved together in base fluids.
		Gum Arabic		
[29]	Al_2_O_3_/W	Ultrasonication: 60 min		- The samples were subject to the sonication process for 60 min. - All samples were found to be stable for up to 10 days.
	SiO_2_/W			
	Al_2_O_3_-SiO_2_/W			
[96]	Al_2_O_3_/W	pH: 5–10	Visual Observation Zeta Potential DLS	- Both nanofluids showed stability for more than one week. - *ξ* Al_2_O_3_: −72.2 mV - *ξ* CuO: −85.1 mV - The value of zeta potential moves away from 0 mV as the amount of SDBS was added to the samples.
	CuO/DI-W	SDBS		
[97]	Al_2_O_3_-SiO_2_/W	Ultrasonication: 4 h P = 100 W *f* = 36 ± 3 kHz	Visual Observation UV-Vis Zeta Potential	- Nanofluids at 0.6 wt.% was stable through observation with little sedimentation, *ξ* = −60.7 mV after 4 weeks - The peak absorbance for 0.6 wt.% nanofluids maintained after 4 weeks
[54]	Al_2_O_3_/EG	Ultrasonication: 2 h	Visual Observation FESEM UV-Vis	- The samples were stable with little sedimentation up to 2 months. - UV-Vis Spectra showed that the absorbance of Al_2_O_3_/EG nanofluids decreased as concentration decreased. - Higher absorption was found at a wavelength between 200 and 400 nm.
		pH: 5.34–5.97		
		pH: Neutralized		

**Table 4:** Summary of thermal conductivity enhancement of various nanofluids.

Author (s)	Nanoparticles		Base Fluids	Size (nm)/Shape		*T* (°C)	Vol.%/wt.%	*k*_enhanced_ (%)	Green
[19]	Al_2_O_3_		BG:W (60:40)	13/spherical		30–80	0.5–2.0 vol.%	T = 80 °C, *ϕ =* 2.0% Max_60: 40_: 13% Max_40: 60_: 24%	Yes
			BW:W (40:60)						
[29]	Al_2_O_3_		water	-		20–50	1.0–3.0 vol.%	T = 20–50 °C *ϕ =* 0.5% Al_2_O_3_ + 2.5% SiO_2_ Max = 17.96–23.61%	No
	SiO_2_						Al_2_O_3_: 0.5 vol.% SiO_2_: 0.5–2.5 vol.%		
	Al_2_O_3_:SiO_2_								
[85]	Al_2_O_3_		EG	13 and 50/spherical		25–50	0.1–1.0 vol.%	T = 50 °C, *ϕ =* 1.0% Max 50 nm: 38%	No
[78]	TiO_2_:SiO_2_	20:80	W:EG (60:40)	TiO_2_: 50/Rod-like SiO_2_: 22/spherical		30–80	1.0 vol.%		No
		40:60							
		50:50							
		60:40							
		80:20							
[18]	SiO_2_		water	40–50/spherical		25–55	0–3.0%	T = 55 °C, *ϕ =* 3.0% Max: 38.2%	Yes
[114]	Activated hybrid carbon/graphene oxide		EG	-		20–40	0.00–0.06 wt.%.	T = 40 °C, *ϕ =* 0.06% Max: 6.47%	Yes
[10]	h-rGO		water	Planar structure		15–45	0.02–0.08 wt.%.	At T = 55 °C and 0.02 < *ϕ* < 0.08, k was enhanced from 8.9 to 35.7%	Yes
[160]	MWCNT: SiC (50:50)		W:EG (50:50)	25–50/ MWCNT: Tubular surface SiC: Almost spherical		61	0–0.75 vol.%	T = 50 °C, *ϕ =* 0.75% Max: 28.86%	No
[21]	TiO_2_		W: BG	80:20	50/spherical	30–80	0.5–2.0 vol.%	T = 80 °C, *ϕ =* 2.0% R_bf_: 80:20 Max: 12.6%	Yes
				70:30					
[84]	Al_2_O_3_		W:EG	40: 60	13/spherical	30–70	0.2–1.0 vol.%	T = 70 °C, *ϕ =* 1.0% R_bf_: 40: 60 Max: 12.8%	No
				50: 50					
				60: 40					
[161]	SiC		water	<100/spherical		22–23.5	0.001, 0.1, 1, 2, 3 vol.%	*ϕ =* 3.0% Max: 7.2%	No
[145]	SiC		water	-		25–60	0.1–3.0 wt.%	T = 60 °C, *ϕ =* 3.0% Max: 2.29%	No
			W:EG (65:35)						
			W: PG (65:35)						
[19]	Al_2_O_3_		BG	13		30–80	0.1–1.0 vol.%	T = 30 °C, *ϕ=* 1.0% Max: 17%	Yes

**Table 5:** Summary of dynamic viscosity behavior of various nanofluids.

Author(s)	Nanofluids	Vol.%/wt.%	*T* (°C)	Size (nm)/Shape	Findings	Green
[115]	TiO_2_/W:EG	0.5–1.5 vol.%	30–70	-	The viscosity decreased from 2.3 to 2.4 times in temperatures between 30 and 70 °C	No
[177]	SiO_2_/W	1.08 vol.% 2.28 vol.%	20–70	Spherical Banana-shaped	The viscosity of banana-shaped SiO_2_ nanoparticles almost similar to the spherical-shaped SiO_2_ nanoparticles	No
	ZnO/W	0.82 vol.% 0.93 vol.%		Polygonal Rod-like	The viscosity of rod-shaped ZnO nanoparticles is less than the polygonal-shaped ZnO nanoparticles	
[115]	TiO_2_/W:EG	0.5–1.5 vol.%	30–80	-	Fluctuation in the relative viscosities in the range of 4.6–33.3% at temperatures between 30 and 80 °C	No
[21]	SiO_2_/W:BG (80:20)	0.5–2.0 vol.%	30–80	22/Spherical	The viscosity increased from 16.02 to 28.9% in the temperature range of 30 to 80 °C at 2.0 vol.%	Yes
	SiO_2_/W:BG (70:30)				The viscosity increased from 17.3 to 37.8% in the temperature range of 30 to 80 °C at 2.0 vol.%	
[171]	C/W:EG (40:60)	0.04–1.0 wt.%	15–60	Nano sphere	The viscosity increased by up to 50% with mass fraction and no significant change with temperature.	Yes
[21]	TiO_2_/W:BG (80:20)	0.5–2.0 vol.%	30–80	50/Spherical	The viscosity increased from 20.5 to 33.8% in the temperature range between 30 and 80 °C at 2.0 vol.%	Yes
	TiO_2_/W:BG (70:30)				The viscosity increased from 29.8 to 53.4% in the temperature range between 30 and 80 °C at 2.0 vol.%	
[178]	rGO/W	1.0–4.0 vol.%	20–70		The rGO/water nanofluids demonstrated a Newtonian behavior. The viscosity decreased from 86.2 to 87.9% between particle concentrations	Yes
[179]	C-MWCNTs/W	0.075–0.175 wt.%.	20–50	-	The viscosity of nanofluids slightly increases from the base fluid.	Yes
[114]	Activated hybrid carbon- graphene oxide/EG	0.00–0.06 wt.%.	20–40	-	The viscosity increased up to 4.16% at 0.06 wt.%	Yes
[180]	TiO_2_-ZnO (70:30)/W:EG	0.1–1.5 vol.%	50–70	TiO_2_ (21) ZnO (10–30)	The viscosity of the hybrid nanofluids increase with the increase in the amount of TiO_2_ nanoparticles	No
	TiO_2_-ZnO (80:20)/W:EG					
	TiO_2_-ZnO (90:10)/W:EG					
[64]	TiO_2_-SiO_2_/W:EG	0.5–3.0 vol.%	30–80	-	The viscosity of the hybrid nanofluids increased by up to 62.5% at 3.0 vol.% and 80 °C	No
[181]	MWCNT- TiO_2_/W:EG	0.05–0.85 vol.%	10–50	-	The maximum increase in viscosity is 83%, found at 0.85 vol.% and 10 °C.	No

## Data Availability

No new data were created or analyzed in this study. Data sharing is not applicable to this article.
